# Multi-Wavelength Narrow-Spacing Laser Frequency Stabilization Technology Based on Fabry-Perot Etalon

**DOI:** 10.3390/mi15101269

**Published:** 2024-10-18

**Authors:** Ju Wang, Ye Gao, Jinlong Yu, Hao Luo, Xuemin Su, Shiyu Zhang, Ruize Zhang, Chuang Ma

**Affiliations:** School of Electrical and Information Engineering, Tianjin University, Tianjin 300072, China; wangju@tju.edu.cn (J.W.); gaoye0610@tju.edu.cn (Y.G.); hello_hluo2020@tju.edu.cn (H.L.); xueminsu@tju.edu.cn (X.S.); zhang_sy1613@tju.edu.cn (S.Z.); zhangruize@tju.edu.cn (R.Z.); machuang@tju.edu.cn (C.M.)

**Keywords:** laser frequency stabilization, Fabry-Perot etalon, DFB-LD, wavelength modulation, digital control

## Abstract

Classical frequency-stabilized lasers have achieved high-frequency stability and reproducibility; however, their extensive wavelength spacing limits their utility in various scenarios. This study introduces a novel frequency-stabilized laser scheme that integrates a Fabry-Perot etalon (FPE) with digital control technology and wavelength modulation techniques. The FPE, characterized by multiple transmission peaks at minimal frequency intervals, provides stable frequency references for different lasers, thereby enhancing the system’s flexibility and adaptability. An error signal is derived from the first-order differentiation of the FPE’s transmission curve. A 180° phase difference was observed in the feedback output signal when the laser’s central frequency diverged from the reference, determining that the direction of the frequency control was accordingly determined.Employing feedback control, the laser’s output frequency is stabilized at the transmission peak frequency of the FPE. Experimental results demonstrate that this stabilization scheme effectively locks the laser’s output wavelength to different transmission peak frequencies of the FPE, achieving 25 GHz wavelength spacing. The frequency stability is improved by two orders of magnitude on a second-level timescale, maintained within hundreds of kHz, equating to a frequency stability level of 10^−10^.

## 1. Introduction

In recent years, laser technology has attracted increasing attention as a crucial tool owing to its various applications in fields such as laser communication [1], dense wavelength division multiplexing (DWDM) [2,3], high-resolution laser spectroscopy [4,5], and precision measurement [6,7]. Distributed feedback semiconductor lasers (DFB-LDs), known for their direct high-speed modulation, low cost, simple structure, and ease of tuning, are attracting increasing attention in laser application research. For instance, in current DWDM systems, typical channel spacings are 50 GHz and 100 GHz [8]. With technological advancements, narrower channel spacings, such as 25 GHz, are utilized in particular ultra-dense wavelength division multiplexing systems [9]. However, the output optical frequency of DFB-LDs is susceptible to frequency drift owing to internal structural instability and external environmental influences, which hinders the reduction of channel spacing in ultra-dense wavelength division multiplexing systems. Additionally, in high-precision laser measurement applications, where the reference for distance measurement is the laser wavelength, frequency drift affects both measurement accuracy and the sensitivity and stability of the measurement system [10]. The stability of the laser wavelength is crucial in phase stability and coherent communication. Phase stability is essential for maintaining the coherence of optical signals, while coherent communication systems rely on precise phase control to achieve efficient data transmission. Frequency drift can lead to phase instability, affecting the signal quality and system bit error rate [11]. Therefore, ensuring the long-term frequency stability of lasers is crucial in various application fields.

Current laser frequency stabilization technology is categorized into passive and active stabilization, with the latter being significantly prevalent [12,13,14,15]. In 2018, Couturier et al. [16] used a commercial wavelength meter to monitor the laser output wavelength in real-time before feeding the measured information back to the laser control system for adjustments, achieving a frequency stability with an absolute Allan deviation of below 10^−9^ over 10 h. However, high-precision wavelength meters, which can only lock the wavelength within a specific range, are costly. In 2020, Zektzer et al. [17] designed a laser wavelength stabilization system using a miniaturized acetylene gas absorption cell, maintaining a frequency stability that exceeded 400 kHz within 34 s for a 1.5 μm laser. In the latest research, Wang Ju et al. [18] developed a low-cost, structurally simple 1.5 μm frequency-stabilized laser system by employing digital control techniques in combination with acetylene gas absorption. Through coarse temperature tuning and fine current tuning via feedback control, the system achieved second-level frequency stability within hundreds of kHz, corresponding to a frequency stability of 10^−10^. Additionally, the laser can operate continuously for over six hours, with long-term frequency stability reaching 10^−9^. Although this system attained a high frequency stability, different vibrational absorption bands of the acetylene gas absorption cell span hundreds of GHz, covering the entire THz range with fixed absorption peaks, making it unsuitable for multi-wavelength narrow-spacing applications. Sharma et al. [19] proposed stabilizing the frequency of an external cavity diode laser (ECDL) using the reference absorption peaks of iodine molecules, achieving a linewidth stability of 0.75 MHz within 100 ms at a laser wavelength of 739.03 nm. However, the iodine molecule absorption spectrum’s wavelength spacing, ranging from a few to several tens of nm, similarly limits its utility for narrow-spacing applications. In 2021, Ding et al. [20] introduced a compact and reliable 589 nm DFB-LD laser frequency stabilization system which locked to the $D_{2a}$ line via the saturated absorption method and achieved a frequency stability of 1.8 MHz over 5000 s. Although effective for locking to this specific fine structure frequency, it restricts the selection of different wavelength frequencies. In 2022, Adamiec et al. [21] developed an active frequency stabilization device for a 1572 nm DFB-LD laser, locking the DFB laser to a selected ${\mathrm{CO}}_{2}$ absorption line and achieving an Allan deviation below 100 kHz within 3 s. In 2024, Wu et al. [22] designed an ECDL active frequency stabilization system based on rubidium atomic saturated absorption spectroscopy, achieving a frequency stability of 10^−10^ and successfully stabilizing the output of a 780 nm laser. Although the gas absorption lines of ${\mathrm{CO}}_{2}$ and rubidium atoms span from several hundred MHz to several tens of GHz, due to the different frequency discrimination curves between each absorption peak, the laser can only be locked to particular frequencies owing to specific frequencies. This limitation hinders achieving effective laser frequency stabilization across different wavelengths in applications requiring multi-wavelength flexibility.

The aforementioned studies indicate that current frequency stabilization schemes using semiconductor lasers as light sources predominantly employ active stabilization techniques based on saturated absorption spectroscopy, significantly enhancing relative stability. Nonetheless, these methods have limitations, including fixed stabilized wavelengths, restricted wavelength spacing ranges, and complex system structures. Consequently, high-precision, multi-wavelength narrow-spacing, and structurally simpler frequency stabilization technologies need to be developed for semiconductor lasers.

The Fabry-Perot etalon (FPE) is a versatile optical device capable of flexibly selecting and stabilizing laser outputs at different wavelengths. Stabilizing laser frequencies using an FPE is analogous to saturated absorption spectroscopy stabilization. Similar to how absorption lines of atoms or gas molecules exhibit peak absorption at the central frequency, the FPE demonstrates optimal transmission at its central frequency. Therefore, the curve of the odd-order derivatives of the transmission intensity can serve as a frequency discrimination curve independent of the wavelength band, rendering it suitable for wavelength selection in laser applications [23,24]. In 2022, Xiaoyuan Zhang et al. [25] proposed an active frequency stabilization system for a single longitudinal mode laser. This system employed an external FPE as the reference and employed an active feedback control mechanism to lock the laser frequency to the transmission peak of the etalon, achieving a frequency stability of 10^−8^. While this method employed an FPE as the frequency stabilization reference to enable fine wavelength tuning, its relative frequency stabilization accuracy was limited to the MHz range and featured a frequency stability of the order of 10^−8^.

This study proposes a high-precision, multi-wavelength narrow-spacing frequency-stabilized laser scheme that integrates an FPE, allowing for the selection of stable frequency values. The system employs fibre-coupled optical components to simplify the optical path and reduce overall size. The FPE features multiple stable transmission peaks with closely spaced frequency intervals. Moreover, its design allows for the realization of different central wavelengths, enabling it to serve as a frequency reference for lasers of various wavelengths. However, gas absorption chambers lock onto particular fine structure frequencies, limiting their capability to achieve wavelength frequency selection. Error signals are generated by performing a first-order derivative calculation on the transmission curve of the FPE. The feedback output signal exhibits a 180° phase shift when the central wavelength deviates from the stabilized reference frequency, facilitating the determination of the control direction for laser frequency. A high-speed comparator enables fine adjustment of the laser’s frequency lock point by setting a threshold, while the digital auxiliary function of the microcontroller unit (MCU) facilitates automatic frequency stabilization at startup, improving the system’s resistance to external environmental fluctuations. A feedback control method involving coarse temperature tuning and fine current tuning is suitable for stabilizing the laser’s output frequency at the transmission peak frequency of the FPE. Experimental results demonstrate that this DFB-LD frequency stabilization scheme achieves high-frequency stability, successfully locking the laser output frequency to different transmission peak frequencies of the FPE with a wavelength spacing of 25 GHz. The frequency stability is improved by two orders of magnitude on a timescale of seconds and maintained within hundreds of kHz, corresponding to a frequency stability level of 10^−10^.

## 2. Experimental Principle

### 2.1. Frequency Identification Properties of the Fabry-Perot Etalon

An ideal FPE consists of two parallel reflecting surfaces. The normalized transmission curve of its transmission spectrum is defined as the ratio of the transmitted light intensity *I* to the incident light intensity $I_{0}$, as expressed in [26].

(1) $$\begin{array}{c} T=\frac{I}{I_{0}}=\frac{\left(1-r_{1}\right)\left(1-r_{2}\right)}{{\left(1-r_{1}r_{2}\right)}^{2}+4\sqrt{r_{1}r_{2}}\sin^{2}\frac{\delta }{2}} \end{array}$$

In Equation (1), $r_{1}$ and $r_{2}$ are the amplitude reflectivities of the two surfaces of the etalon and δ is the phase difference between two adjacent beams participating in the multiple-beam interference within the etalon. Assuming the intensity reflectivities of each surface are $R_{1}$ and $R_{2}$, respectively, and $r_{1}=r_{2}$, $R_{1}=R_{2}=R=r_{1}^{2}$, the phase difference δ is a function of the optical frequency and can be expressed as follows [27]:

(2) $$\delta =\omega \times \frac{2nl}{c}$$

where *n* is the refractive index of the medium between the two reflecting surfaces of the etalon, *c* is the cavity thickness, and *l* is the speed of light. The normalized transmission curve of the etalon can be expressed as

(3) $$T\left(\omega \right)=\frac{1}{1+\frac{4R}{{\left(1-R\right)}^{2}}sin^{2}\frac{\omega nl}{c}}$$

The first derivative curve of the normalized transmission curve T(ω) of the transmission spectrum is

(4) $$T^{\prime }\left(\omega \right)=-\frac{4nlR{\left(1-R\right)}^{2}\sin\frac{2\omega nl}{c}}{c{\left[{\left(1-R\right)}^{2}+4Rsin^{2}\frac{\omega nl}{c}\right]}^{2}}.$$

In the multi-wavelength narrow-spacing laser frequency stabilization system proposed in this study, the FPE acts as an external reference for frequency stabilization. Assuming the material and coatings of the etalon are of high optical quality, the absorption loss within the FPE is insignificant. However, any residual absorption will slightly reduce the finesse (Q factor) of the etalon, affecting the sharpness of the transmission peaks, broadening them, and slightly decreasing the sensitivity of the wavelength determination. We used an etalon with anti-reflection coatings and low absorption in our experiments to mitigate this effect, ensuring high finesse and sensitivity in wavelength determination. Additionally, diffraction losses, which arise mainly from imperfections or deviations in the surface flatness and parallelism of the etalon, can reduce peak transmittance and slightly broaden the resonance peaks, affecting the accuracy and sensitivity of wavelength locking. Therefore, we ensured that the FPE used in our setup has a high-quality surface flatness to minimize the impact of diffraction losses on the finesse of the etalon to reduce the impact of this issue. Initially, experimental tests and theoretical analyses were conducted to examine the frequency discrimination characteristics of the FPE transmission peaks, facilitating precise tuning and control of the laser frequency.

**Figure 1:**
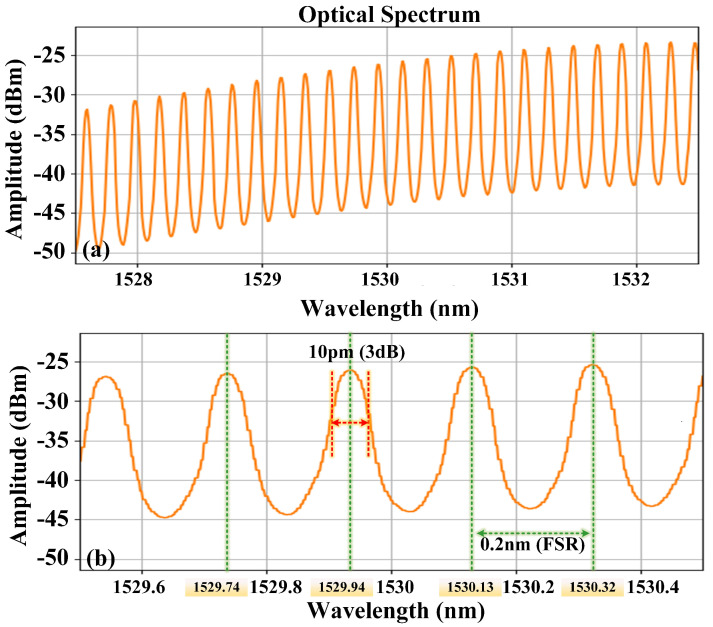
Distribution of the Fabry–Perot etalon transmission peaks near 1530 nm: (**a**) Span: 5 nm (**b**) Span: 1 nm.

The light source for these experiments was the amplified spontaneous emission (ASE) light from an erbium-doped fibre amplifier (EDFA) which was injected into the FPE. The FPE transmission peak spectrum was recorded at its output. Figure 1a depicts the transmission peak spectrum distribution near 1530 nm. These peaks are distinctly separated and act as references for laser frequency stabilization. In this study, transmission peaks with centre wavelengths of 1529.74 nm, 1529.94 nm, 1530.13 nm, and 1530.32 nm were selected as frequency stabilization references. The 3 dB bandwidth of the transmission peaks for the FPE utilized in this study is nominally approximately 10 pm, with a free spectral range (FSR) of approximately 0.2 nm (25 GHz). Figure 1b illustrates the detailed measured spectrum of these transmission peaks. Consequently, the minimum wavelength spacing for frequency stabilization using the FPE is approximately 0.2 nm.

**Figure 2:**
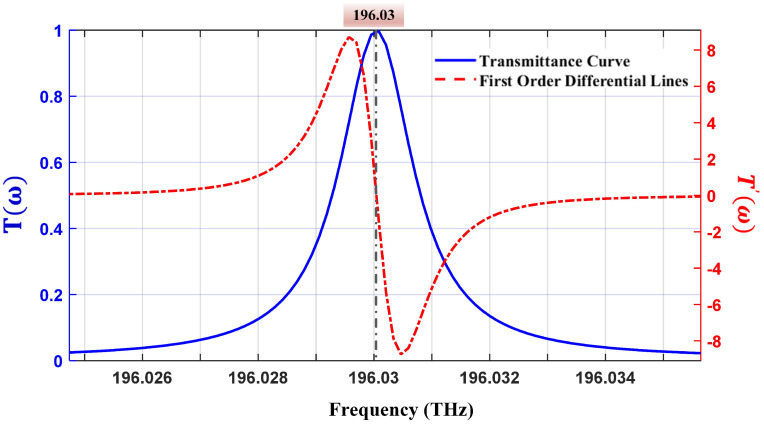
Simulated schematic of the Fabry−Perot etalon transmission spectrum and its first derivative curve.

Figure 2 illustrates the simulated schematic of the normalized transmission spectrum of the FPE (Equation (3)) alongside its first derivative curve (Equation (4)). The normalized transmission spectrum T(ω) of the FPE is depicted by the blue solid line, while the red dashed line represents its first derivative curve. The blue line indicates the central frequency of the transmission peak at 196.03 THz (1529.94 nm). Observations from the red line indicate that the first derivative curve is approximately linear in the region near the central frequency of the transmission peak. The first derivative is negative when the laser frequency exceeds the central frequency of the transmission peak and positive when it is below the central frequency, which facilitates frequency discrimination.

### 2.2. Generation of Error Signals

In the approximately linear region of the first derivative curve of the FPE transmission spectrum, the first derivative is often utilized as a frequency discrimination curve. The modulation–demodulation method, a widely employed technique, generates the error signal [28]. By applying frequency modulation to the laser, a sinusoidal modulation signal is introduced to the system:

(5) $$\Delta \omega_{t}=Ksin\Omega t$$

where *K* is the amplitude of the modulation signal, Ω is the angular frequency of the modulation signal and the modulated laser frequency oscillates sinusoidally around the central frequency:

(6) $$\omega =\omega_{0}+\Delta \omega_{t}=\omega_{0}+Ksin\Omega t$$

where $\omega_{0}$ is the central frequency of the laser. The normalized transmission spectrum obtained after the frequency-modulated laser passes through the FPE can be expanded using the Taylor series.

(7) $$\begin{array}{cc} T\left(\omega \right) & =T\left(\omega_{0}\right)+T^{\prime }\left(\omega_{0}\right)Ksin\Omega t\\ & +\frac{1}{2}T^{\prime \prime }\left(\omega_{0}\right)K^{2}sin^{2}\Omega t+\cdots \end{array}$$

The optical signal that passes through the FPE is multiplied by a reference signal MsinΩt (which is in-phase and has the same frequency as the modulation signal) after being detected by a photodetector.

(8) $$\begin{array}{cc} T(\omega )\cdot Msin\Omega t & =T\left(\omega_{0}\right)Msin\Omega t+T^{\prime }\left(\omega_{0}\right)KMsin^{2}\Omega t\\ \; & +\frac{1}{2}T^{\prime \prime }\left(\omega_{0}\right)K^{2}Msin^{3}\Omega t+\cdots \\ \; & =\frac{1}{2}[T^{\prime }\left(\omega_{0}\right)KM\\ \; & -T^{\prime }\left(\omega_{0}\right)KMcos2\Omega t]\\ \; & +T(\omega_{0})Msin\Omega t\\ \; & +\frac{1}{2}T^{\prime \prime }\left(\omega_{0}\right)K^{2}Msin^{3}\Omega t+\cdots \end{array}$$

Among the components at the output of the mixer, only $T^{\prime }\left(\omega_{0}\right)\mathrm{KM}$ is a DC term. However, other components include the frequency Ω or its harmonics. By passing this signal through a low-pass filter (with a cutoff frequency significantly lower than Ω), the $T^{\prime }\left(\omega_{0}\right)\mathrm{KM}$ term, which is the suitable error signal, can be isolated. As derived in Section 2.1, when the laser frequency exceeds the central frequency of the FPE transmission peak, i.e., $T^{\prime }\left(\omega_{0}\right)<0$ is negative, resulting in a negative error signal. Conversely, when the laser frequency is below the central frequency of the FPE transmission peak, i.e., $T^{\prime }\left(\omega_{0}\right)>0$, the error signal is positive. This error signal provides feedback and corrects the laser output frequency, thereby achieving laser frequency stabilization.

**Figure 3:**
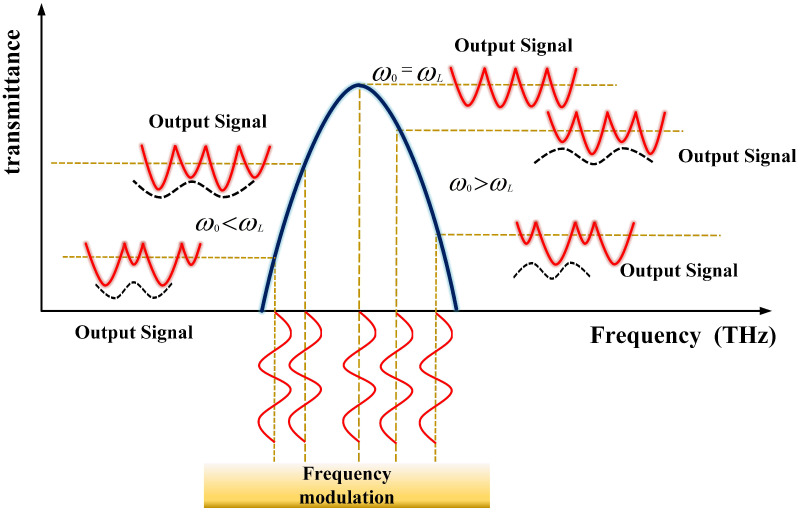
Output signal of the laser when the central frequency of the output is at different positions relative to the absorption peak.

Figure 3 illustrates the output signal of the laser when the central frequency of the laser is at various positions relative to the FPE transmission peak. The central frequency of the laser produces different output signals depending on its position relative to the transmission peak of the FPE. Here, $\omega_{0}$ represents the central frequency of the laser and $\omega_{L}$ represents the central frequency of the FPE transmission curve. When the central frequency of the laser aligns precisely with the central frequency of the FPE transmission curve ($\omega_{0}=\omega_{L}$), the laser frequency exhibits a sinusoidal oscillation with a period of 2Π/Ω. Owing to the symmetry of the cosine function regarding the central frequency of the laser and the symmetric frequency variations relative to the FPE transmission curve, the frequency modulation of Ω yields an output signal with a modulation frequency of 2Ω. This signal exhibits a completely flat amplitude without any Ω frequency components.

When the central frequency of the laser is below the central frequency of the FPE transmission curve ($\omega_{0}<\omega_{L}$), the symmetry of the FPE transmission curve relative to the laser frequency change is disrupted. This asymmetry produces an output signal with alternating amplitudes and an envelope featuring a repetition frequency of Ω. As the average wavelength gradually deviates further from the FPE centre wavelength, this shift weakens the 2 Ω frequency signal component and strengthens the Ω frequency signal component. The degree of deviation from the FPE centre wavelength determines the Ω frequency signal to the 2 Ω frequency signal component. Conversely, a similar phenomenon occurs but with a critical distinction when the central frequency of the laser exceeds that of the FPE ($\omega_{0}>\omega_{L}$). Owing to the opposite direction of the frequency shift, the phase of the Ω frequency component signal differs by 180º. Therefore, the output signal can exhibit two distinct phase states depending on the direction of the central frequency shift, indicating different shift directions.

This analysis underpins our approach to stabilizing the laser frequency by leveraging the characteristics of the laser frequency modulation.By identifying the position of the laser output frequency relative to the FPE transmission peak, we can effectively control and stabilize the laser’s output wavelength through feedback mechanisms.

## 3. Experimental Setup

**Figure 4:**
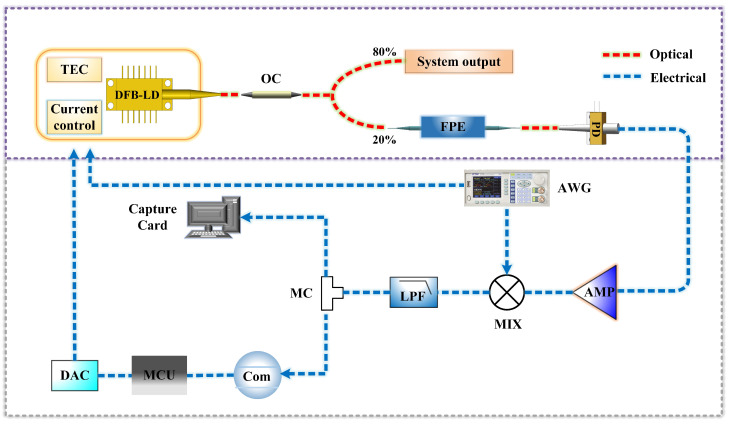
Schematic of the laser frequency stabilization system structure. DFB-LD: distributed feedback semiconductor Laser, OC: optical coupler, FPE: Fabry-Perot etalon, PD: photodetector, AMP: amplifier, Mix: mixer, AWG: arbitrary waveform generator, LPF: low-pass filter, MC: microwave coupler, Com: high-speed comparator, MCU: microcontroller unit, DAC: digital-to-analogue converter.

Figure 4 depicts a simple experimental setup for frequency stabilization of a digitally controlled multi-wavelength, narrow-spacing DFB laser. The red dashed lines illustrate the optical signals transmitted within the optical fibres, while the blue dashed lines represent electrical signals. The light source is a DFB-LD with a linewidth of 1 MHz and an output power of 10 mW. The output pigtail of the laser diode (LD) comprises a polarization-maintaining (PM) fibre and the output wavelength is tenable within a range of 1530 nm ± 2 nm. The optical field is split by a coupler with an 80%:20% splitting ratio, where 80% of the light serves as the output light source of the system and the remaining 20% is directed toward feedback correction in the frequency stabilization system and enters the FPE.

The light that passes through the FPE is converted into an electrical signal by a photodetector (PD) equipped with a fibre pigtail input, a 3 dB bandwidth of 14 GHz, a maximum input optical power of 17 dBm and a responsivity of 0.8 A/W. Subsequently, the electrical signal is processed by analogue circuitry, where it is amplified using an operational amplifier (OPA1611), a single-ended input op-amp characterized by an input voltage noise of 1.1 nV$\sqrt{\mathrm{Hz}}$ at 1 kHz, owing to its ultra-low noise and distortion properties. A signal generator emits two in-phase signals at the same frequency: a 12 MHz sine wave with a peak-to-peak value of 200 mV and a DC offset of 0 mV, which modulates the optical frequency of the laser by affecting the current source driving the laser. Simultaneously, another 12 MHz sine wave (with a peak-to-peak value of 1.5 V and a DC offset of 0 V) is mixed with the amplified signal from the photodetector in a multiplier (AD835) for demodulation. The mixed signal is filtered through a low-pass filter (LPF) to isolate a DC error signal indicative of the frequency offset.

A high-speed data acquisition card (Queentext, QT114) captures the DC error signal, facilitating the observation of the feedback control effect. The error signal is detected using a high-speed comparator (ADCMP602) circuit and paired with an MCU. In the frequency stabilization system design, the high-speed comparator circuit detects the error signal generated by the laser’s frequency deviation from the centre frequency of the FPE transmission peak. When the input error signal is lower than the threshold voltage set by the comparator, it outputs a low level. Conversely, when it exceeds the threshold, it outputs a high level. Based on the level received from the comparator circuit, the MCU determines the direction of the laser’s frequency deviation relative to the centre frequency of the FPE transmission peak. Additionally, it adjusts the output voltage of a digital-to-analogue converter (DAC8830, 16-bit data input, single power supply, with the swiftest response time of 1 μs), modifying the code value with the smallest step size to perform feedback control on the laser temperature and current regulation modules, ensuring a stable frequency output from the laser.

## 4. Experimental Results

### 4.1. Automatic Frequency Stabilization After Power on

**Figure 5:**
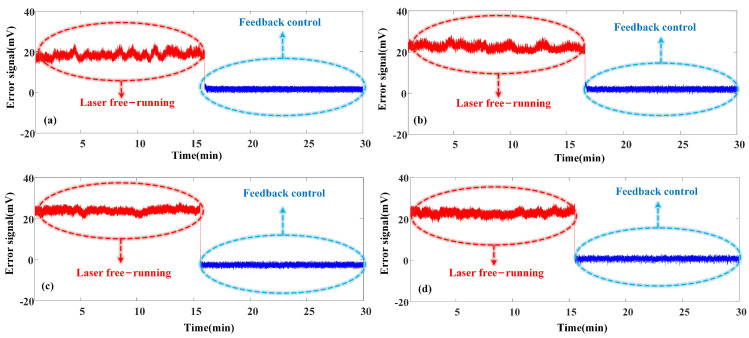
Error signal of the laser during free operation and feedback control at different frequency stabilization reference points: (**a**) 1529.74 nm, (**b**) 1529.94 nm, (**c**) 1530.13 nm, (**d**) 1530.32 nm.

Based on Figure 2 and Equations (4) and (8), when the laser output frequency coincides with the centre frequency of the FPE transmission peak, the theoretical value of the error signal should be 0 mV. The threshold voltage of the comparator was set to a positive value of 1 mV to facilitate detection. In cases where the feedback control system is inactive and the laser operates freely, the frequency drift of the laser induces instability in the output laser’s centre frequency, leading to irregular fluctuations in the error signal. By adjusting the laser’s operating temperature, the wavelength of the DFB-LD was fine-tuned to approximately 1529.74 nm, 1529.94 nm, 1530.13 nm, and 1530.32 nm, respectively. However, these settings were not perfectly aligned. Figure 5a–d illustrates the initial half of the measurement, where the centre wavelengths of the FPE transmission peaks at 1529.74 nm, 1529.94 nm, 1530.13 nm, and 1530.32 nm, respectively, served as the frequency stabilization reference. This segment depicts the DC error signal fluctuations when the laser operates freely. After activating the feedback control system, the MCU starts to evaluate the relative position of the laser output frequency to the centre frequency of the FPE transmission peak based on the high/low levels received from the comparator. Subsequently, it adjusts the output voltage of the digital-to-analogue converter, effectively managing the feedback control of the laser’s temperature and current regulation modules. This adjustment causes the error signal to rapidly approach the threshold voltage until it stabilizes near this threshold, achieving frequency stabilization in the laser. However, once the feedback system is activated, the previously irregular error signal fluctuations are swiftly moderated to near 0 mV, as shown in Figure 5a–d. This adjustment indicates the successful implementation of laser frequency stabilization control.

**Figure 6:**
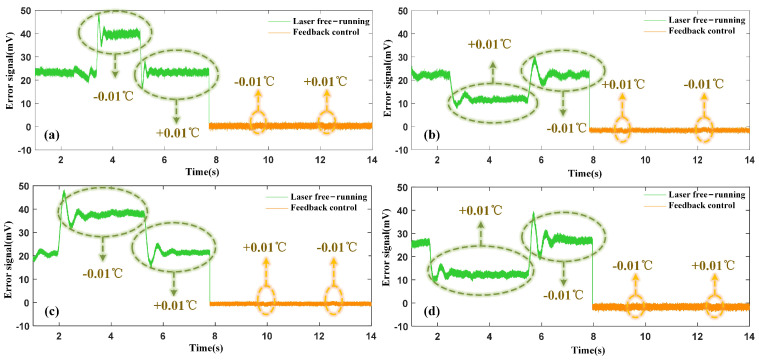
Impact of changing the laser operating temperature on the error signal at different frequency stabilization reference points: (**a**) 1529.74 nm (**b**) 1529.94 nm (**c**) 1530.13 nm (**d**) 1530.32 nm.

The operating temperature of the laser was deliberately varied (±0.01 °C) during its free operation to evaluate the stability of the frequency stabilization system. This induced significant fluctuations in the laser frequency, which corresponded to large variations in the error signal, as depicted by the green line in Figure 6a–d. When the frequency stabilization system was activated, the error signal was effectively locked near the threshold voltage. Subsequently, when the laser’s operating temperature was again varied (±0.01 °C), any resulting fluctuations in the error signal were promptly re-locked near the threshold by the feedback control system, illustrated by the yellow line in Figure 6a–d. These dynamic experimental results demonstrate the efficacy of the stabilization system, demonstrating that the laser’s output frequency can be consistently stabilized at different centre frequencies of the FPE transmission peaks. The system compensates for frequency deviations caused by temperature changes and maintains suitable stability across these variations, demonstrating its robustness in real-world operating conditions.

### 4.2. Frequency Stability Estimation

The performance of the frequency stabilization system can be assessed through a straightforward estimation method. This approach involves analysing the amplitude of fluctuations in the feedback error signal and integrating these data with the frequency discrimination sensitivity of the system. The fluctuations in the laser frequency can be assumed, which facilitates the calculation of the system’s frequency stability [29,30].

**Figure 7:**
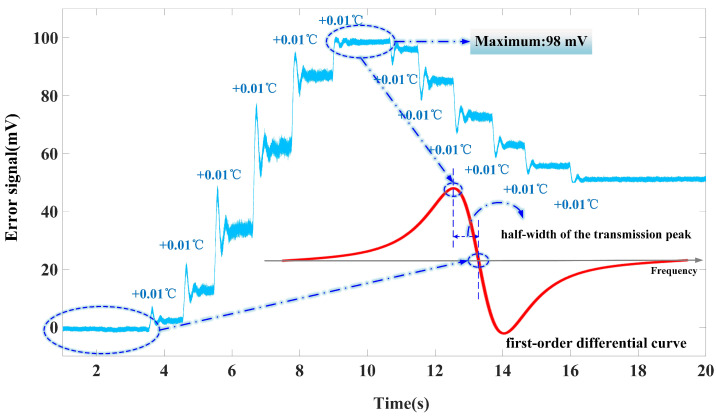
Schematic diagram of the maximum feedback DC signal.

The voltage signal output after passing through the low-pass filter serves as the error signal, indicating the degree of deviation of the laser’s output frequency from the centre frequency of the FPE transmission peak. Based on Equation (8) and the first derivative of the transmission spectrum depicted in Figure 2, the feedback DC signal is 0 mV when the laser output frequency aligns exactly with the centre frequency of the transmission peak. When the laser output frequency shifts to the left side of the transmission peak’s centre frequency, the feedback DC signal reaches its maximum value. The frequency discrimination sensitivity is determined by the slope of the first derivative signal at the zero crossing point. Owing to the approximately linear behaviour of the first derivative curve within the 3 dB linewidth (10 pm) of the FPE, when the laser’s output frequency is centred at the peak, adjustments in the laser’s operating temperature that maximize the amplitude of the feedback DC signal corresponding to a frequency are offset to an equivalent of half the linewidth of the transmission peak, $\Delta f_{m}$. Therefore, the frequency discrimination slope of the FPE transmission peak can be defined as $S_{f}$:

(9) $$\begin{array}{c} S_{f}=\frac{\Delta V_{DC}}{\Delta f_{m}} \end{array}$$

where $\Delta V_{DC}$ represents the change in the feedback DC signal output by the system. The half-width $\Delta f_{m}$ of the FPE transmission spectrum is 625 MHz. When the laser output frequency deviates from the transmission peak by this half-width, the amplitude of the error signal signal is maximised. The maximum detected value of the error signal is 98 mV, as shown in Figure 7. Thus, the frequency discrimination slope $S_{f}$ of the FPE is 0.16mV/MHz. The stability of the laser output frequency, $\sigma_{f}$, is defined as follows:

(10) $$\begin{array}{c} \sigma_{f}=\frac{\delta_{e}}{S_{f}f_{0}} \end{array}$$

where $\delta_{e}$ represents the fluctuation amplitude of the error signal and $f_{0}$ is the center frequency of the laser output. Therefore, the stability of the laser’s output frequency can be inferred by measuring the voltage stability of the error signal.

**Figure 8:**
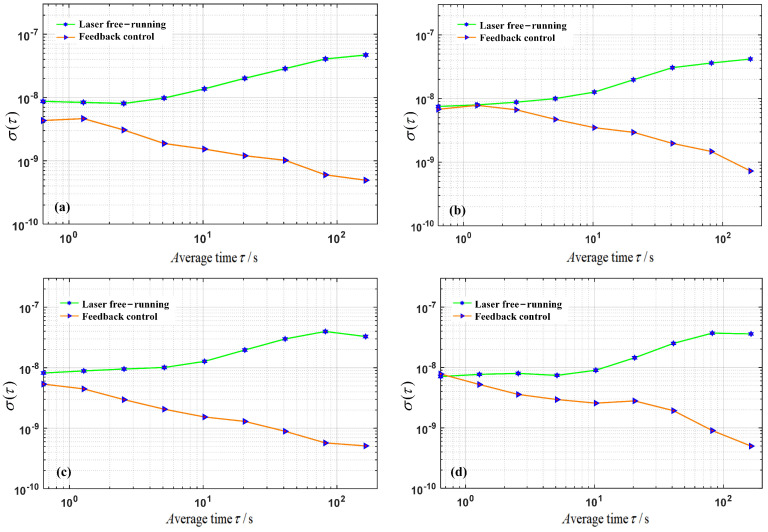
Frequency stability of the laser during free operation and feedback control at different frequency stabilization reference points: (**a**)1529.74 nm (**b**) 1529.94 nm (**c**) 1530.13 nm (**d**) 1530.32 nm.

The Allan variance $\sigma^{2}$ of the error signal data was measured and calculated for both free operation and feedback control, exceeding 100 ms–100 s averaging times to assess the stability of the laser frequency. The laser’s second-level frequency stability was subsequently determined using Equation (10), as illustrated in Figure 8. In Figure 8a–d, the green lines in the frequency stability of the laser during free operation increases with the system operating time, achieving stability at the 10^−8^ level. Conversely, the red lines show that, when employing centre frequencies of 1529.74 nm, 1529.94 nm, 1530.13 nm, and 1530.32 nm as frequency stabilization references (with a frequency spacing of approximately 25 GHz), the frequency stability after feedback control decreases as the system operating time increases. At an integration time of 100 s, the stability of the laser after feedback control is maintained at the 10^−10^ level. The calculated second-level frequency stability is within hundreds of kHz, representing a reduction of two orders of magnitude compared with the free operation, thus yielding over a hundredfold improvement in stability and demonstrating a significant frequency stabilization effect.

In terms of frequency stability limitations, the finesse of the FPE plays a critical role in laser wavelength stabilization. Higher finesse results in sharper transmission peaks, enhancing wavelength locking precision. However, any defects, such as surface roughness or deviations in parallelism, reduce finesse and broaden the transmission peaks, decreasing wavelength stabilization accuracy. In this experiment, we employed optimized high-quality etalons with precision coatings and surface flatness to ensure high finesse and reliable wavelength stabilization. From the electrical aspect, wavelength stability and amplitude noise are limited by both the control precision and response speed of the feedback circuit. Accurate modulation of frequency is critical, for which we employed a high-precision DAC controller. Additionally, we improved the system’s response speed by using a high-response DAC chip. If the feedback loop is designed with a slower response, the system may fail to effectively suppress amplitude fluctuations, leading to increased noise. By optimizing both control precision and response speed, we enhanced system performance, ensuring reliability in the wavelength stabilization.

## 5. Conclusions

This paper presents a high-precision, multi-wavelength, narrow-spacing, low-cost, and structurally simple frequency-stabilized laser scheme that incorporates an FPE. This system allows for the selection of stable frequency values. The system integrates all optical components as fibre-optic input and output types. This configuration simplifies the optical path and reduces the system’s size. The FPE features multiple transmission peaks with very small frequency intervals, and it can be designed to achieve different central wavelengths, allowing it to serve as a frequency stabilization reference for lasers operating at various wavelengths. In contrast, gas absorption cells typically can only lock onto specific fine structure frequencies, limiting their capability in regard to wavelength frequency selection. This design enhances the system’s flexibility and adaptability for various applications. The study results demonstrated that this DFB-LD frequency stabilization scheme offers high frequency stability, locking the laser output frequency to the transmission peaks of different FPEs with a minimum wavelength interval of 25 GHz. The second-level frequency stability improves by two orders of magnitude, stabilizing within hundreds of kHz and achieving a frequency stability level of 10^−10^. We employed optimised high-quality standards to ensure coating precision and surface flatness, achieving high finesse and reliable wavelength stability. On the circuit side, we further enhanced the system’s performance by using high-precision and high-response DAC chips, ensuring the reliability of wavelength stabilization.

## Data Availability

The raw data supporting the conclusions of this article will be made available by the authors on request.
